# Alarming situation of emerging H5 and H7 avian influenza and effective control strategies

**DOI:** 10.1080/22221751.2022.2155072

**Published:** 2022-12-12

**Authors:** Jianzhong Shi, Xianying Zeng, Pengfei Cui, Cheng Yan, Hualan Chen

**Affiliations:** a Guangdong Laboratory for Lingnan Modern Agriculture, Guangzhou, People’s Republic of China; b State Key Laboratory of Veterinary Biotechnology, Harbin Veterinary Research Institute, CAAS, Harbin, People’s Republic of China

**Keywords:** Review, evolution, spread, vaccination, H5 and H7 avian influenza

## Abstract

Avian influenza viruses continue to present challenges to animal and human health. Viruses bearing the hemagglutinin (HA) gene of the H5 subtype and H7 subtype have caused 2634 human cases around the world, including more than 1000 deaths. These viruses have caused numerous disease outbreaks in wild birds and domestic poultry, and are responsible for the loss of at least 422 million domestic birds since 2005. The H5 influenza viruses are spread by migratory wild birds and have caused three waves of influenza outbreaks across multiple continents, and the third wave that started in 2020 is ongoing. Many countries in Europe and North America control highly pathogenic avian influenza by culling alone, whereas some countries, including China, have adopted a “cull plus vaccination” strategy. As the largest poultry-producing country in the world, China lost relatively few poultry during the three waves of global H5 avian influenza outbreaks, and nearly eliminated the pervasive H7N9 viruses that emerged in 2013. In this review, we briefly summarize the damages the H5 and H7 influenza viruses have caused to the global poultry industry and public health, analyze the origin, evolution, and spread of the H5 viruses that caused the waves, and discuss how and why the vaccination strategy in China has been a success. Given that the H5N1 viruses are widely circulating in wild birds and causing problems in domestic poultry around the world, we recommend that any unnecessary obstacles to vaccination strategies should be removed immediately and forever.

Influenza A viruses are important pathogens that continually challenge both human and animal health. The genome of influenza A virus comprises eight gene segments: basic polymerase 2 (PB2), basic polymerase 1 (PB1), acidic polymerase (PA), hemagglutinin (HA), nucleoprotein (NP), neuraminidase (NA), matrix (M), and nonstructural protein (NS). Each of these segments encodes one to three proteins. On the basis of the antigenicity of the HA and NA proteins, influenza viruses are divided into different subtypes. Currently, 16 HA subtypes and nine NA subtypes have been detected in avian species. H1N1, H2N2, and H3N2 viruses have caused four influenza pandemics since 1918, and H1N1 and H3N2 viruses continue to co-circulate in humans globally. Viruses of several other subtypes that circulate in animals have also jumped to humans on multiple occasions [1–8], and some of them have shown pandemic potential [9–12].

The avian influenza viruses are maintained and circulate in wild birds. Although different subtypes of viruses have been detected in domestic poultry, especially waterfowl that come into close contact with wild birds, only three HA subtypes—H5, H7, and H9—have spread and been detected in domestic poultry across wide geographic areas. Some strains bearing the HA gene of the H5 or H7 subtypes are highly pathogenic for poultry and have caused severe problems for the global poultry industry. In this review, we briefly summarize the H5 and H7 influenza outbreaks and the damage they have caused to the global poultry industry and public health, analyze the evolution and spread of H5 viruses, and discuss the effectiveness of the poultry vaccination strategy for highly pathogenic avian influenza control.

## Avian influenza outbreaks caused by H5 viruses

In the last century, avian influenza outbreaks caused by different H5 viruses have occurred in eight countries or regions. The first recorded highly pathogenic avian influenza outbreak was caused by H5N1 virus in chickens in Scotland in 1959 [13]; in 1966, an avian influenza outbreak in turkeys in Canada was caused by H5N9 virus [14]; an H5N2 virus caused multiple outbreaks in chickens and turkeys in the US from 1983 to 1985 [15]; in 1983, an H5N8 virus caused disease outbreaks in turkeys, chickens, and ducks in Ireland [16]; in 1991, an H5N1 virus caused a disease outbreak in turkeys in England [17]; an H5N2 virus caused multiple outbreaks in chickens and turkeys in Mexico from 1994 to 1995 [18]; and in 1997, an H5N1 virus and an H5N2 virus caused outbreaks in chickens in Hong Kong and Italy, respectively [19,20].

**Figure 1.**
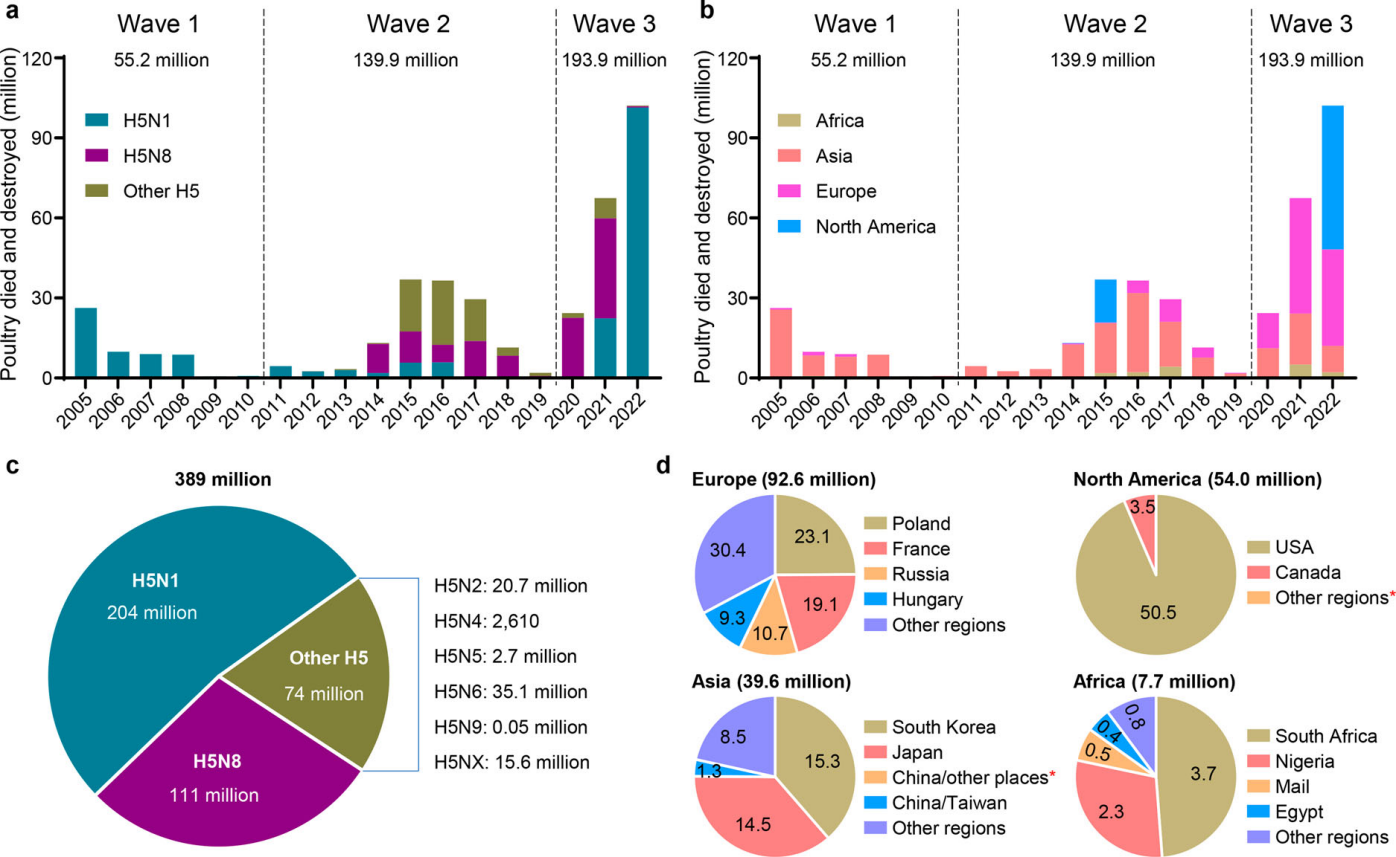
Damage caused to the global poultry industry since 2005 by different H5 avian influenza viruses based on information reported in the OIE-World Animal Health Information System. The number of poultry that died or were destroyed during outbreaks caused by different subtypes of H5 influenza viruses (a, c) in different continents (b), and (d) the number of poultry that died or were destroyed in different countries or regions since 2020. *, fewer than 10,000 birds died or were destroyed in the indicated country or regions.

In this century, the first H5 avian influenza outbreak occurred in Hong Kong in 2002, caused by an H5N1 virus [20]. In 2003 and 2004, avian influenza outbreaks caused by H5N1 viruses were reported in several Asian countries, including Vietnam, Thailand, Indonesia, China, Japan, South Korea, Cambodia, and Lao [21]. The number of poultry lost in the outbreaks that occurred before 2004 is not available; however, between January 2005 and November 2022, H5 highly pathogenic avian influenza viruses have caused 8534 outbreaks and the loss of 389 million poultry around the world (Figure 1(a)), according to the information reported in the OIE-World Animal Health Information System (OIE-WAHIS, https://wahis.woah.org). The viruses caused three waves of outbreaks in multiple countries in Asia, Africa, Europe, and North America. The first wave, which occurred from 2005–2010, was caused by H5N1 viruses and 55.2 million poultry died or were destroyed. The outbreaks that occurred during this period were mainly reported in Asian countries, although some African and European countries were also affected (Figure 1(b)). The second wave, which occurred from 2011 to 2019, was caused by multiple subtypes of H5 viruses and 139.9 million poultry died or were destroyed (Figure 1(a)). The outbreaks in this period were reported in Asia, Europe, Africa, and North America (Figure 1(b)). The third wave started in 2020 and was mainly caused by H5N8 and H5N1 viruses; 193.9 million poultry died or were destroyed as of the end of November 2022 (Figure 1(a)). The outbreaks in this period were mainly reported in Europe and North America, although some were also reported in Asian and African countries (Figure 1(b)).

Of the 389 million poultry that died or were destroyed, H5N1 viruses were responsible for 204 million, H5N8 viruses were responsible for 111 million, and the other 74 million poultry losses were caused by other H5 viruses (Figure 1(c)). Of note, 92.6 million, 54 million, 39.6 million, and 7.7 million poultry died or were destroyed in Europe, North America, Asia, and Africa, respectively, since 2020 (Fig 1(d)). The large number of birds that died or were destroyed in the third wave in a relatively short period of time suggests that the ongoing third wave will be much more serious than previous ones, if control measures taken in Europe and North America do not change.

## Avian influenza outbreaks caused by H7 viruses

In the last century, avian influenza outbreaks caused by different H7 viruses have occurred in five countries. The first outbreak was caused by H7N3 virus in turkeys in England in 1963 [22]. Five outbreaks occurred in domestic poultry in Australia in 1976, 1985, 1992, 1994, and 1997, respectively, and were caused by H7N7 virus (1975 and 1985), H7N3 virus (1992 and 1994), and H7N4 virus (1997) [23–25]. In 1979, H7N7 virus caused outbreaks in domestic poultry in Germany and England [26]; in 1995, an H7N3 virus caused outbreaks in chickens in Pakistan [27]; and in 1999–2000, H7N1 virus caused outbreaks in multiple species of domestic poultry in Italy [28].

In 2002, outbreaks in chickens caused by H7N3 virus occurred in Chile [29]; in 2003, outbreaks in different domestic poultry caused by H7N7 virus occurred in The Netherlands, Belgium, and Germany [30]; and in 2004, an H7N3 virus caused outbreaks in chickens in Canada [31]. The number of birds killed or destroyed in the outbreaks that occurred before 2004 is not available.

Between January 2005 and November 2022, different H7 highly pathogenic avian influenza viruses caused 106 outbreaks and the loss of over 33 million poultry around the world, according to data reported in the OIE-WAHIS (Table 1). These outbreaks occurred in 10 countries across Asia, Europe, North America, and Oceania, including Australia, Canada, Mexico, the US, the Democratic People's Republic of Korea, China, the UK, Spain, Italy, and Germany. Of note, 77 outbreaks in countries in North America were caused by H7N3 viruses, resulting in the loss of more than 29 million birds. The H7N7 viruses caused 10 outbreaks in European countries and the Democratic People's Republic of Korea, and H7N9 viruses caused outbreaks in the US and China. At least three different subtypes of H7 viruses were responsible for the outbreaks in Australia. These facts indicate that the H7 viruses are actively circulating in nature and continue to pose a threat to the global poultry industry.

**Table 1.** Outbreaks caused by H7 highly pathogenic avian influenza viruses between January 2005 and November 2022 around the world.

Year	Continent	Country	Subtype	Outbreaks	Number of poultry dead/destroyed
2005	Asia	Democratic People's Republic of Korea	H7N7	3	218,788
2007	North America	Canada	H7N3	1	49,100
2008	Europe	United Kingdom	H7N7	1	25,000
2009	Europe	Spain	H7N7	1	308,640
2012–2022	North America	Mexico	H7N3	75	29,813,496
2012	Oceania	Australia	H7Nx*	1	50,000
2013	Europe	Italy	H7N7	2	1,178,861
2013	Oceania	Australia	H7N2	1	490,000
2015	Europe	Germany	H7N7	1	10,104
2015	Europe	United Kingdom	H7N7	1	179,865
2016	Europe	Italy	H7N7	1	66,972
2016	North America	United States	H7N8	1	43,500
2017–2018	Asia	China	H7N9	12	745,665
2017	North America	United States	H7N9	1	74,000
2020	North America	United States	H7N3	1	34,160
2020	Oceania	Australia	H7N7	3	435,378
Total				106	33,723,529

*The NA subtype was not reported.

## Human infections caused by H5 and H7 viruses

In 1997, H5N1 avian influenza viruses transmitted from birds to humans in Hong Kong causing the deaths of 6 of 18 infected persons [3]; this was the first report of human infection with lethal H5N1 virus and attracted wide attention. Since 2003, 865 human cases of H5N1 virus infection have been reported in more than 20 countries across Asia, Africa, Europe, and North America (Table 2). Seventy-five human cases of H5N6 virus infection have been reported in China and Lao, whereas seven human cases of H5N8 virus infection were reported in Russia (Table 2). Among the 947 human cases involving different viruses reported from 2003 to April 2022, 488 were fatal (Table 2). Studies have identified several key amino acids in the HA of H5N1 viruses that increase the affinity of these viruses for human-type receptors [10,11,32], and several research groups have demonstrated that H5N1 virus can become transmissible via respiratory droplets in ferrets or guinea pigs after obtaining certain mutations or reassorting with human influenza viruses [10–12].

**Table 2.** Human infections caused by H5 viruses around the world from January 2003 to April 2022*.

Country	Case information				
	Total Number	Year	Virus subtype	Number infected	Number of fatalities
Azerbaijan	8	2006	H5N1	8	5
Bangladesh	8	2008, 2011–2013, 2015	H5N1	8	1
Cambodia	56	2005–2014	H5N1	56	37
Canada	1	2013	H5N1	1	1
China	127	2003, 2005–2015	H5N1	53	31
		2014–2022	H5N6	74**	32
Djibouti	1	2006	H5N1	1	0
Egypt	359	2006–2017	H5N1	359	120
India	1	2021	H5N1	1	1
Indonesia	200	2005–2015, 2017	H5N1	200	168
Iraq	3	2006	H5N1	3	2
Lao	4	2007, 2020	H5N1	3	2
		2021	H5N6	1	0
Myanmar	1	2007	H5N1	1	0
Nepal	1	2019	H5N1	1	1
Nigeria	1	2007	H5N1	1	1
Pakistan	3	2007	H5N1	3	1
Russia	7	2020	H5N8	7	0
Thailand	25	2004–2006	H5N1	25	17
Turkey	12	2006	H5N1	12	4
UK	1	2021	H5N1	1	0
US	1	2022	H5N1	1	0
Viet Nam	127	2003–2005, 2007-2010, 2012–2014	H5N1	127	64
Total	947	/	/	947	488

*Data obtained from the WHO website.

**Forty-nine of the 75 human cases infected with H5N6 virus have occurred since January 2021.

As described above, different subtypes of H7 highly pathogenic influenza viruses have caused disease outbreaks in poultry around the world. Historically, both low and highly pathogenic H7 influenza viruses caused human infections, and a total of 1687 human cases were documented in eight countries between 1959 and 2019 (Table 3). The cases reported in Australia, Canada, Italy, Mexico, the UK, and the US ranged from one to 10, and all of the infected individuals survived the infection (Table 3) [5,7,8,33–43]. Eighty-nine human cases infected with highly pathogenic H7N7 virus were reported in The Netherlands in 2003, and one veterinarian died from the infection [6]. One human case infected with H7N4 virus and 1568 human cases infected with H7N9 viruses were reported in China [4]; 616 of the H7N9 virus infections were fatal (Table 3).

**Table 3.** Human infections caused by H7 viruses around the world since 1959.

Country	Case information					
	Total Number	Time period	Virus subtype	Pathotype	Number infected	Number of fatalities
Australia	1	1977	H7N7	Highly pathogenic (HP)	1	0
Canada	2	2004	H7N3	HP	2	0
China	1569	Feb. 2013–Sept, 2017	H7N9	Low pathogenic (LP)/HP	1564	615
		Oct. 2017–Sept, 2018	H7N9	HP	3	1
		2018	H7N4	LP	1	0
		2019	H7N9	HP	1	0
Italy	10	2002–2003	H7N3	LP	7^#^	0
		2013	H7N7	HP	3	0
Mexico	2	2012	H7N3	HP	2	0
The Netherlands	89	2003	H7N7	HP	89	1
UK	6	1996	H7N7	LP	1	0
		2006	H7N3	LP	1	0
		2007	H7N2	LP	4	0
US	8	1959	H7N7	HP	1	0
		1979	H7N7	LP	4	0
		2002–2003, 2016	H7N2	LP	3	0
Total	1687	/	/	/	1687	617

^#^ Serologic evidence only.

The thousands of human cases of infection with H5 or H7 viruses indicate that humans are highly susceptible to these viruses. Epidemiology studies have shown that humans become infected mainly through exposure to virus-infected poultry or a contaminated environment [44]; human-to-human transmission has been very limited. Therefore, before the H5 and H7 viruses acquire the ability to transmit from human to human, control of these viruses in animals is essential and effective to prevent them from infecting humans.

## Evolution and spread of H5 viruses by migratory wild birds

Influenza viruses evolve mainly through the accumulation of mutations in their genomes and reassortment between different strains. The HA genes of H5 viruses detected since 2003 can be roughly divided into nine different clades, and some clades have been further divided into different subclades [45]. Viruses bearing the HA gene of the same clade may have different NA and internal genes, and may therefore belong to different genotypes. Most H5 viruses have been detected only in certain countries or regions, and only strains that infect long-distance migratory birds have spread over different continents and caused disastrous consequences. Of the four large-scale intercontinental transmissions of H5 viruses in the past 20 years, two originated in Asia (H5N1 in 2005 and H5N8 in 2014) and two originated in Europe (H5N8 in 2020 and H5N1 in 2021).

The first H5 virus that was widely spread by migratory wild birds was the so-called Qinghai Lake-like H5N1 virus. In May 2005, migrating bar-headed geese carrying at least three different genotypes of H5N1 virus bearing the clade 2.2 HA gene flew over the Himalayas to the egg island in Qinghai Lake in western China, a major breeding ground for migratory birds [21,46]. The viruses spread to several other species on the island, including great black-headed gulls, brown-headed gulls, great cormorants, ruddy shelducks, and whooper swans, and caused the death of over 6,000 wild birds at the lake from 4 May to 29 June 2005 [21]. The viruses were subsequently spread by whooper swans to Mongolia and Russia in August 2005, and were then widely detected in wild birds and domestic poultry in European and African countries in 2006 [47]. These viruses were eradicated in China and many other European countries in a relatively short time, but they circulated in poultry for many years and caused severe disease in poultry and humans in Egypt [48].

The second H5 virus that was widely spread by migratory birds was the H5N8 virus bearing the subclade 2.3.4.4 HA gene. In early 2014, a novel H5N8 virus bearing the subclade 2.3.4.4 HA gene caused multiple outbreaks in migratory birds and domestic ducks in South Korea [49], and was subsequently spread to Europe, North America, and East Asia by migratory birds [50]. The H5N8 viruses continued to evolve and spread and caused numerous outbreaks in wild birds and domestic poultry in countries in Asia, Europe, and Africa [51]. Although similar H5N8 viruses were also detected in swans and grey-legged geese in China at the end of 2016 and in earlier 2017 [52], they did not infect and spread among domestic poultry in China, probably because the vaccine used in poultry in China was effective against these H5N8 viruses [53].

The third H5 virus that was spread widely by migratory birds was the H5N8 virus bearing the subclade 2.3.4.4b HA gene. In January 2020, a novel H5N8 virus bearing the clade 2.3.4.4b HA caused outbreaks in chickens in Poland and then started a new wave of outbreaks in poultry and wild birds in countries in Europe, Africa, and Asia [54]. By the end of March 2022, the H5N8 viruses were reported in more than 42 countries, and nearly 60 million domestic poultry had died or were destroyed (http://empres-i.fao.org/eipws3 g/). The HA genes of these H5N8 viruses formed two different branches that probably separated in early 2018 [55]. The viruses with the branch I HA circulated in domestic poultry and wild birds in Poland, Hungary, Germany, and Czech Republic in the spring and summer of 2020, and were then detected in domestic poultry and wild birds in Japan and Korea in the winter of that year. In January 2021, a virus bearing the branch I HA was detected in a whopper swan in Shandong Province, China. The virus bearing the branch II HA was first detected in chickens in Iraq in May 2020, then caused multiple disease outbreaks in domestic poultry in July and August 2020 in Russia, and was responsible for subsequently widespread disease outbreaks in wild birds and domestic poultry in Russia and many countries in the Middle East, Europe, Africa, and Asia. The virus bearing the branch II HA began to be detected in swans and other wild birds in China from October 2020, and was also detected in ducks and geese in 2021. Of note, H5N8 viruses bearing the branch II HA gene were also detected in humans in Russia and in seals and a fox in the UK [56–58].

## Emergence, evolution, and global dissemination of the recent H5N1 influenza viruses bearing the clade 2.3.4.4b HA

During their circulation in nature, the H5N8 viruses reassorted with other influenza viruses and generated several other subtypes of H5 viruses that bear the clade 2.3.4.4b HA gene. H5N2 viruses were detected in wild birds in Serbia and domestic poultry in Taiwan, China and Bulgaria [54,59]; H5N3 viruses were detected in wild birds in Denmark, France, Germany, Ireland, and The Netherlands [54]; H5N4 viruses were detected in wild birds in Germany, The Netherlands, and Sweden [54]; H5N5 viruses were detected in wild birds and domestic poultry in Iran and many countries in Europe [54]; and H5N6 viruses were detected in ducks in China and have caused multiple cases of human infection [2,60]. The most important descendant of the H5N8 virus is the novel H5N1 virus that was first detected in The Netherlands [61]. Unlike the H5N2, H5N3, H5N4, H5N5, and H5N6 viruses, each of which has only been detected in countries on one or two continents, the H5N1 virus bearing the clade 2.3.4.4b HA gene took over from H5N8 virus and started the fourth large-scale, intercontinental spread. This novel H5N1 virus has caused 4,284 disease outbreaks, as of the end of March 2022, in many countries in Europe, Africa, Asia, and the Americas since it emerged in The Netherlands in October 2020 [54].

**Figure 2.**
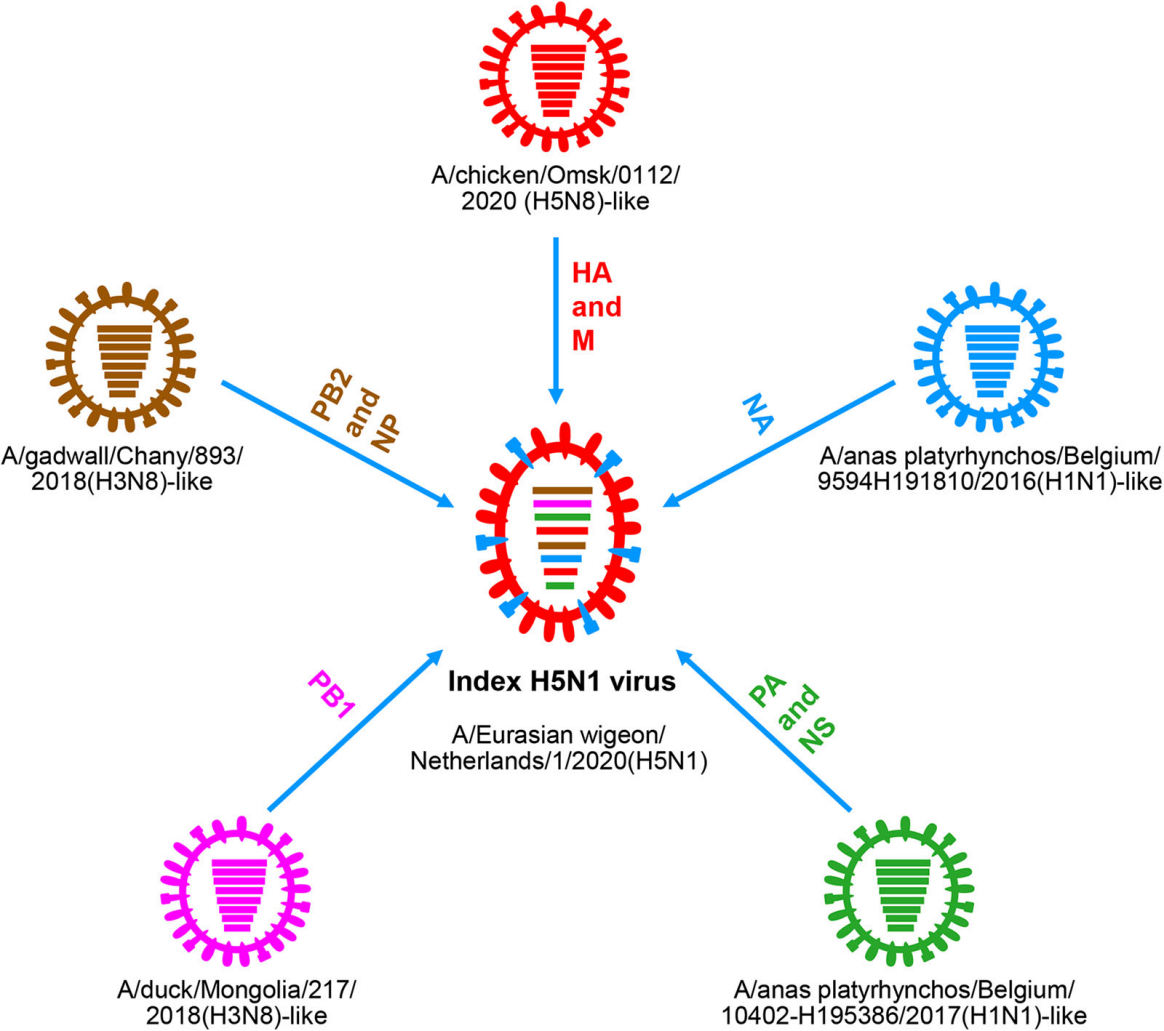
Formation of the index H5N1 virus bearing the 2.3.4.4b HA gene in 2020. The eight bars represent the eight gene segments (from top to bottom: PB2, PB1, PA, HA, NP, NA, M, and NS), and the colour of the bar indicates the closest donor strain of the gene segment.

Genetic analysis revealed that the novel index H5N1 virus A/Eurasian wigeon/Netherlands/1/2020(H5N1) is a reassortant of five different viruses: an H5N8 virus provided the HA and M genes, an A/gadwall/Chany/893/2018(H3N8)-like virus provided the PB2 and NP genes, an A/duck/Mongolia/217/2018(H3N8)-like virus provided the PB1 gene, an A/anas platyrhynchos/Belgium/10402-H195386/2017(H1N1)-like virus provided the PA and NS genes, and an A/anas platyrhynchos/Belgium/9594H191810/2016 (H1N1)-like virus provided the NA gene (Figure 2) [62].

**Figure 3.**
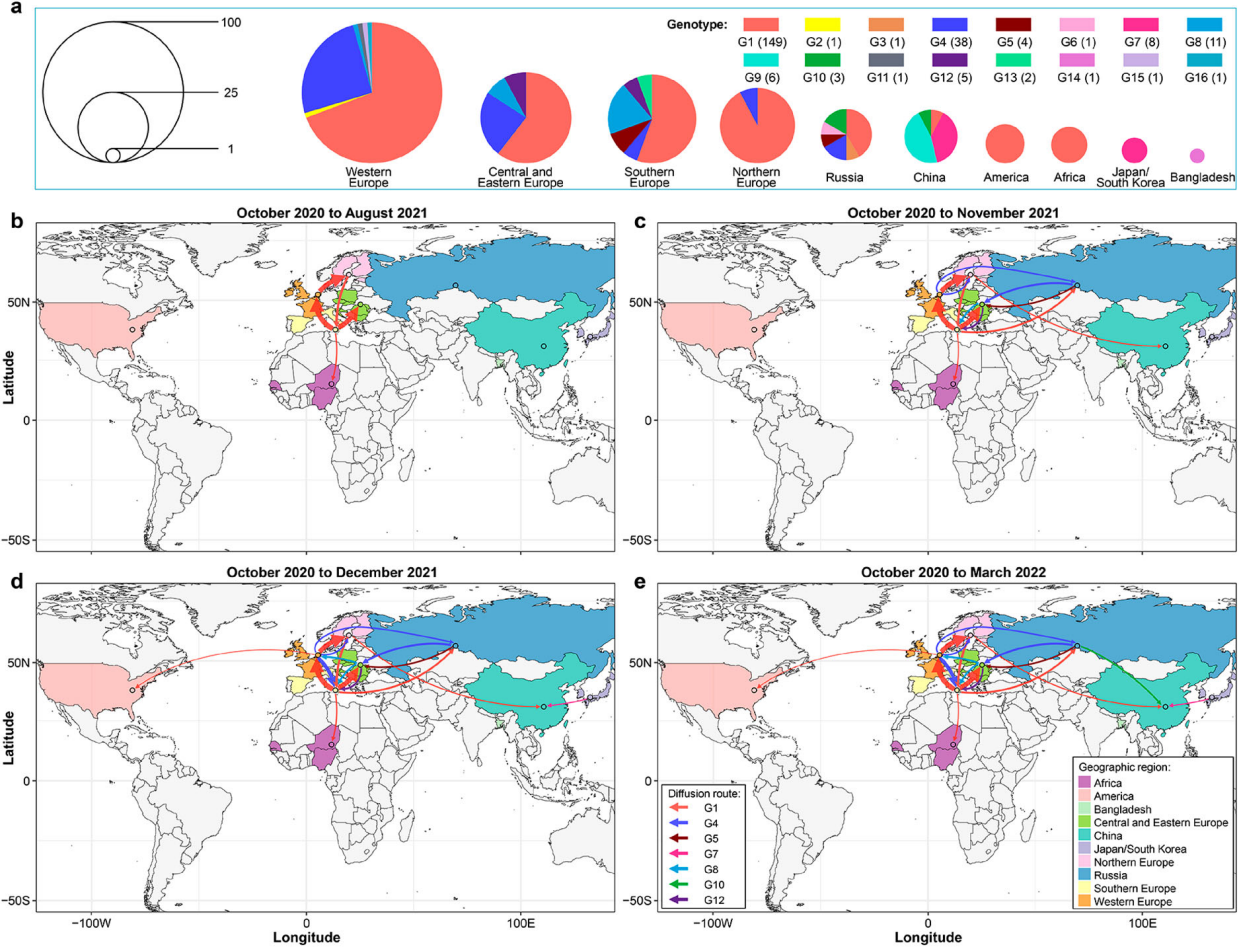
Spatiotemporal spread of H5N1 viruses bearing the clade 2.3.4.4b HA gene. (a) Genotype and distribution of 233 H5N1 viruses isolated from 28 countries between October 2020 and March 2022. (b–d). Emergence and spread of the indicated seven genotypes that were detected in more than one country/region/continent.

Cui et al. analyzed 233 representative H5N1 viruses bearing the clade 2.3.4.4b HA gene detected in Europe, Africa, Asia, and North America that were reported from October 2020 to March 2022 and revealed their spatiotemporal spread. They found that these viruses formed 16 different genotypes (G1-G16) (Figure 3(a)), and viruses of nine genotypes were only detected in one country or one region (Europe was analyzed as four regions—Northern Europe, Southern Europe, Central and Eastern Europe, and Western Europe – in Figure 3 of this review): G2, G11, G15, and G16 were only detected in Western Europe, G13 was only detected in Central and Eastern Europe, G3 and G6 were only detected in Russia, and G9 and G14 were detected in China and Bangladesh, respectively. Viruses of the other seven genotypes spread between countries, regions, or continents (Figure 3(b–e)). Between October 2020 and August 2021, the G1 virus circulated in multiple countries in Europe and Africa (Figure 3(b)); between August 2021 and November 2021, viruses of the G4, G5, G8, and G12 genotypes spread among European countries, and the G1 virus spread from southern Europe to Russia and from northern Europe to China (Figure 3(c)). In the following month, the G1 virus spread from Western Europe to the US, and the G7 virus was generated in Japan/Korea and spread to China (Figure 3(d)). The G10 virus was generated and detected first in Russia in October 2021 and then spread to and was detected in China in March 2022 (Figure 3(e)). Of note, H5N1 viruses are still circulating in multiple countries and causing disease outbreaks in wild birds and poultry [54], and continued reassortment and spread of the viruses are inevitable.

## Control of H5 influenza by vaccination: the China experience

Different countries have adopted different strategies to control highly pathogenic avian influenza. Many countries in Europe and North America control highly pathogenic influenza by culling infected and suspected birds (also called the stamping-out strategy), whereas some countries, including China, have adopted a “cull plus vaccination” strategy.

Over 17 billion poultry, including 4 billion ducks, are reared annually in China. Many birds, especially ducks and geese, are often reared in open fields with no biosecurity measures. We started to develop an H5 vaccine as soon as the first highly pathogenic H5N1 virus was detected in Guangdong in 1996 [63]. In addition to the inactivated vaccine described below, a novel Newcastle virus (NDV)-vectored H5 avian influenza bivalent live vaccine has been used in chickens in China since 2006 [64], and the first H5 DNA vaccine was approved in 2018 [65], but has not been used yet due to vaccine updates. A duck enteritis virus (DEV)-vectored bivalent live vaccine has been constructed and found to provide fast and complete protection in ducks against H5N1 avian influenza virus and highly lethal duck enteritis virus [66]. Most importantly, the DEV-vectored vaccine provided good cross-protection against challenge with different clades of viruses [67]. The DEV-vectored vaccine is not yet officially used to control avian influenza in ducks, as its licence is still pending.

An inactivated vaccine produced with the naturally isolated H5N2 low pathogenic virus A/turkey/England/N28/73(H5N2) was used in China from 2004 to 2006. However, influenza virus mutates easily, and mutation of the HA gene often causes antigenic variation. The biggest challenge for the vaccination strategy is ensuring that the vaccine matches the circulating virus. To address this challenge, a platform for generating vaccine seed viruses by using reverse genetics was established, and an ideal vaccine seed virus containing the modified HA gene and native NA gene of a prevalent H5 virus and the internal genes of the high-growth A/Puerto Rico/8/1934 (H1N1) (PR8) virus can be generated within a week.

Similar to the introduction of H5N8 and H5N1 viruses into China over the past two years, viruses carrying different clades or subclades of HA genes have been introduced into China over the past two decades [52,55,62,68–70]. In response, since 2004, ten different H5 seed viruses generated by reverse genetics have been used for inactivated vaccine production to control and eliminate these viruses (Table 4). Unlike the NDV-vectored vaccines, which are used only in chickens, the reverse genetics inactivated vaccines have been used in chickens and waterfowl, and their effectiveness in these species is well documented [64,71]. The H5-Re1 vaccine seed virus, which derives its HA and NA genes from A/goose/Guangdong/1/1996 (H5N1), started to be used in 2004 and provided solid protection against viruses bearing the clade 0 HA, clade 1 HA, clade 2.2 HA, or 2.3.4 HA gene [68,72,73]. In March 2008, the H5-Re1 seed virus was replaced by the H5-Re5 seed virus, which derived its HA and NA genes from A/duck/Anhui/1/2006(H5N1). The H5N1 viruses bearing the clade 2.3.4 HA gene were eliminated by using the H5-Re5 vaccine and use of the vaccine was suspended in June 2012 (Table 4). The H5-Re4 and H5-Re7 vaccine seed viruses were developed in 2006 and 2014, respectively, to control viruses bearing the clade 7.2 HA gene, which were completely eliminated in China in 2017 (Table 4) [74]. The H5-Re6 and H5-Re12 vaccine seed viruses were developed and used in 2012 and 2018, respectively, to control viruses bearing the clade 2.3.2 HA gene and viruses bearing the clade 2.3.2.1f HA gene, respectively; the use of these vaccines was stopped in 2017 and 2021, respectively, when the viruses were eliminated in China (Table 4) [75,76]. The H5-Re8, H5-Re11, H5-Re13, and H5-Re14 vaccine seed viruses were developed to control H5 viruses bearing different subclades of 2.3.4.4 HA that have been introduced into China in recent years, and currently only the H5-Re13 and H5-Re14 vaccines are used to control the local H5 virus bearing clade 2.3.4.4h HA and the globally circulating H5 viruses bearing clade 2.3.4.4b HA (Table 4) [53,76,77]. Of note, the vaccine used in China is updated when a clear antigenic difference between the vaccine and the newly detected virus is observed, even though sometimes the vaccine could still provide complete protection against the emerging virus.

**Table 4.** Inactivated vaccine seed viruses generated by reverse genetics for the control of highly pathogenic avian influenza in China since 2004^a^.

Seed virus (subtype)	HA donor virus (clade)^b^	Application period^c^	Effective against influenza virus of a different subtype (clade)	Reference
H5-Re1 (H5N1)	GS/GD/1/1996(H5N1) (0)	03/2004–03/2008	H5 (0, 1, 2.2, 2.3.4)	[68,72,73]
H5-Re4 (H5N1)	CK/SX/2/2006(H5N1) (7.2)	07/2006–04/2014	H5 (7.2)	[76]
H5-Re5 (H5N1)	DK/AH/1/2006(H5N1) (2.3.4)	03/2008–06/2012	H5 (2.3.4)	[76]
H5-Re6 (H5N1)	DK/GD/S1322/2010(H5N1) (2.3.2)	06/2012–09/2017	H5 (2.3.2)	[75]
H5-Re7 (H5N1)	CK/LN/S4092/2011(H5N1) (7.2)	04/2014–09/2017	H5 (7.2)	[74]
H5-Re8 (H5N1)	CK/GZ/4/2013(H5N1) (2.3.4.4g)	12/2015–12/2018	H5 (2.3.4.4g)	[53]
H5-Re11 (H5N1)	DK/GZ/S4184/2017(H5N6) (2.3.4.4h)	12/2018–12/2021	H5 (2.3.4.4h)	[76]
H5-Re12 (H5N1)	CK/LN/SD007/2017(H5N1) (2.3.2.1f)	12/2018–12/2021	H5 (2.3.2.1f)	[76]
H5-Re13 (H5N6)	DK/FJ/S1424/2020(H5N6) (2.3.4.4h)	01/2022–	H5 (2.3.4.4h)	[77]
H5-Re14 (H5N8)	WS/SX/4-1/2020(H5N8) (2.3.4.4b)	01/2022–	H5 (2.3.4.4b)	[77]
H7-Re1 (H7N9)	PG/SH/S1069/2013(H7N9)	09/2017–12/2018	H7N9	[33,34]
H7-Re2 (H7N9)	CK/GX/SD098/2017(H7N9)	12/2018–07/2020	H7N9	[76,82]
H7-Re3 (H7N9)	CK/IM/SD010/2019(H7N9)	07/2020–12/2021	H7N9	[77]
H7-Re4 (H7N9)	CK/YN/SD024/2021(H7N9)	01/2022–	H7N9	[77]

^a^ Only vaccine seed viruses prepared by the Harbin Veterinary Research Institute are listed in this table.

^b^ Abbreviations: GS, goose; CK, chicken; DK, duck; WS, whooper swan; PG, pigeon; GD, Guangdong; SX, Shanxi; AH, Anhui; LN, Liaoning; GZ, Guizhou; FJ, Fujian; SH, Shanghai; GX, Guangxi; IM, Inner Mongolia; YN, Yunnan.

^c^ When two or three seed viruses are used at the same time, it means that these seed viruses are used for bivalent or trivalent inactivated vaccine production.

## Emergence, evolution, and effective control of H7N9 influenza virus in China

**Figure 4.**
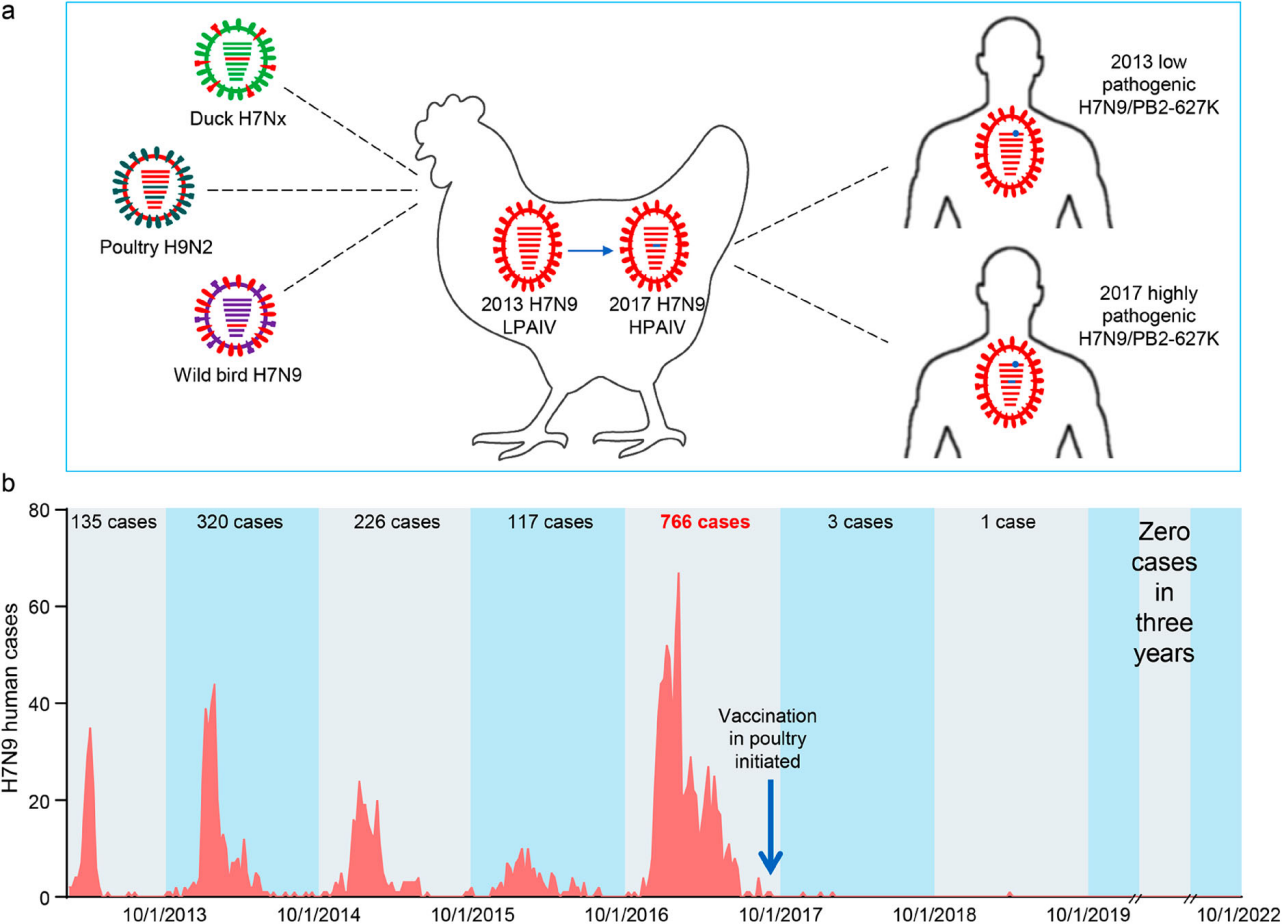
H7N9 viruses detected in China and the human infections they have caused since 2013. (a) Diagram of the emergence and evolution of H7N9 viruses in China. LPAIV, low pathogenic avian influenza virus; HPAIV, highly pathogenic avian influenza virus. (b) Human infections with H7N9 viruses in China.

In February 2013, the H7N9 virus emerged in the live poultry markets in China. Genetically the H7N9 virus is a reassortant of three different viruses: A/duck/Zhejiang/12/2011(H7N3)-like virus provided the HA gene, A/wild bird/Korea/A14/2011(H7N9)-like virus provided the NA gene, and the local H9N2 viruses provided the six internal genes (Figure 4(a)) [78]. Animal studies indicated that the early H7N9 viruses were low pathogenic in chickens and hardly infected ducks [79]; however, after four years of circulation in nature, the viruses obtained certain amino acids in their HA cleavage site and became highly pathogenic in chickens in Guangdong in 2017 (Figure 4(a)) [9]. The increased replicative ability of highly pathogenic H7N9 viruses enabled them to reassort with other duck viruses and generate novel lethal H7 viruses in ducks [33].

The H7N9 viruses have high potential to cause a human influenza pandemic. The H7N9 viruses bind to human-type receptors with high affinity and to avian-type receptor with very low affinity [9,79], which allows the virus to infect humans very easily, as evidenced by the fact that the virus caused over 1560 human infections in five waves from February 2013 to 30 September 2017, with a mortality rate of nearly 40%. Between 1 October 2016 and 30 September 2017, there were 766 human cases (48.9% of the total) reported [33,34], which raised concerns that an even large number of human infections may occur in the subsequent wave. Sequence analysis indicated that after replication in humans, over 78% of the H7N9 strains acquired the 627K mutation in their PB2 gene [9], and Liang and colleagues found that the low polymerase activity attributed to the viral PA protein is the intrinsic driving force behind the emergence of PB2 627K during H7N9 virus replication in mammals [80]. This PB2 627K mutation dramatically increases the replication and virulence of H7N9 virus in mammals, and promotes the respiratory droplet transmission of the H7N9 viruses in mammalian animal models [9]. A recent study indicated that efficient replication of H7N9-PB2/627K virus in the lungs of mice activates gasdermin E-mediated pyroptosis in alveolar epithelial cells and triggers a lethal cytokine storm in mice, thereby revealing the underlying mechanism behind the lethality of H7N9 virus infection in humans [81].

The H7N9 viruses not only caused severe public health problems and concerns, but also caused considerable damage to the poultry industry in China. During each human H7N9 infection wave, in addition to culling poultry from poultry markets that were positive for the virus, tons of uninfected poultry and poultry products were destroyed because people were afraid to consume them. The highly pathogenic H7N9 virus that emerged in early 2017 caused several disease outbreaks on chicken farms in many provinces [33]. Given the damage the H7N9 lethal virus has and will cause to poultry and the high risk it poses to human health, control and eradication of both the low and highly pathogenic H7N9 viruses became the highest priority for animal disease control authorities in China in 2017. Five waves of human infections combined with the emergence of the highly pathogenic H7N9 virus suggested that stamping-out was not a successful measure for H7N9 control; therefore, a vaccination strategy was developed.

To save labour and increase the efficiency of the poultry vaccine strategy, an H5/H7 bivalent inactivated vaccine was developed by using the H7N9-Re1 and H5-Re8 viruses as seed viruses (Table 4) [33,34]. The vaccine was extensively evaluated for safety and efficacy in the laboratory setting with different H5 and H7 viruses. The vaccine provided solid protection against the H7N9 low pathogenic virus and different H7N9 highly pathogenic viruses in chickens [33,34], and its application in poultry was initiated in September 2017 in China. The prevalence of H7N9 virus in poultry was largely prevented, as evidenced by the fact that the isolation rate of H7N9 virus in poultry was reduced by 93.3% after birds were inoculated with the H5/H7 vaccine [33]. More importantly, the vaccination of poultry successfully eliminated human infections with H7N9 virus: only three human cases and one human case were reported during the sixth and seventh waves, respectively, and no human case has been detected since April 2019 (Figure 4(b)).

The H7N9 viruses has still occasionally been detected in chickens, and the vaccine seed virus has been updated three times (Table 4) [76,77,82]. One study indicated that the recent H7N9 viruses have lost the ability to bind to human-type receptors [82], suggesting that the risk to public health posed by the recent viruses may be reduced compared with the earlier ones.

In summary, H5 and H7 subtype avian influenza viruses have caused severe problems to the global poultry industry with more than 389 million domestic birds dying or being destroyed since 2005, including 193.9 million birds lost between January 2020 and November 2022. These viruses also pose severe threats to public health and have caused 2634 human cases with over 1000 fatalities. Vaccines have been used in poultry to successfully prevent highly pathogenic influenza virus infection in China; even though the globally circulating H5 viruses have been detected in many species of wild birds and occasionally in ducks or geese in recent years, they have never caused problems on routinely vaccinated poultry farms in China, and the pervasive H7N9 viruses have been nearly eliminated in China. H5N1 viruses bearing the clade 2.3.4.4b HA gene are widely circulating in wild birds and causing problems in domestic poultry in numerous countries around the world. To improve animal welfare, reduce economic damage, and reduce human infections, vaccination should be immediately and seriously considered as a control strategy not only in underdeveloped countries, but also in developed countries. Any unnecessary obstacles to vaccination strategies should be removed immediately and forever.
